# An Affective Neuroscience Framework for the Molecular Study of Internet Addiction

**DOI:** 10.3389/fpsyg.2016.01906

**Published:** 2016-12-16

**Authors:** Christian Montag, Cornelia Sindermann, Benjamin Becker, Jaak Panksepp

**Affiliations:** ^1^Institute of Psychology and Education, Ulm UniversityUlm, Germany; ^2^Key Laboratory for NeuroInformation/Center for Information in Medicine, School of Life Science and Technology, University of Electronic Science and Technology of ChinaChengdu, China; ^3^Department of Integrative Physiology and Neuroscience, College of Veterinary Medicine, Washington State UniversityPullman, WA, USA

**Keywords:** ANPS, primary emotional systems, Internet addiction, digital addiction, Panksepp, GPIUS-2, personality, smartphone addiction

## Abstract

Internet addiction represents an emerging global health issue. Increasing efforts have been made to characterize risk factors for the development of Internet addiction and consequences of excessive Internet use. During the last years, classic research approaches from psychology considering personality variables as vulnerability factor, especially in conjunction with neuroscience approaches such as brain imaging, have led to coherent theoretical conceptualizations of Internet addiction. Although such conceptualizations can be valuable aid, the research field is currently lacking a comprehensive framework for determining brain-based and neurochemical markers of Internet addiction. The present work aims at providing a framework on the molecular level as a basis for future research on the neural and behavioral level, in order to facilitate a comprehensive neurobiological model of Internet addiction and its clinical symptomatology. To help establish such a molecular framework for the study of Internet addiction, we investigated in *N* = 680 participants associations between individual differences in tendencies toward Internet addiction measured by the Generalized Problematic Internet Use Scale-2 (GPIUS-2) and individual differences in primary emotional systems as assessed by the Affective Neuroscience Personality Scales (ANPS). Regression analysis revealed that the ANPS scales FEAR and SADNESS were the ANPS scales most robustly positively linked to several (sub)scales of the GPIUS-2. Also the scales SEEKING, CARE and PLAY explain variance in some of the GPIUS-2 subscales. As such, these scales are negatively linked to the GPIUS-2 subscales. As the ANPS has been constructed on substantial available brain data including an extensive molecular body with respect to evolutionary highly conserved emotional circuitry in the ancient mammalian brain, the present study gives first ideas on putative molecular mechanisms underlying different facets of Internet addiction as derived from associations between tendencies toward Internet addiction and individual differences in primary emotional systems. For example, as SADNESS is linked to the overall GPIUS-2 score, and the neuropeptide oxytocin is known to downregulate SADNESS, it is conceivable that the neuropeptide might play a role in Internet addition on the molecular level. Our findings provide a theoretical framework potentially illuminating the molecular underpinnings of Internet addiction. Finally, we also present data on the ANPS and smartphone addiction at the end of the paper. Similar to the reported associations between the ANPS and the GPIUS-2, these correlations might provide an initial outline for a framework guiding future studies that aim to address the molecular basis of smartphone addiction.

## Introduction

The Internet has dramatically changed the way we live, finding our way easily into unknown territory, communicating efficiently with our loved ones, and facilitating professional connects, thereby promoting collaborative science with investigators around the globe. According to Internetworldstats (Internetworldstats, 2016,^1^) participation rates for Internet usage were 49.2% in June 2016, ergo half the world population had access to the Internet at present. Despite many advantages due to the digital revolution, many scienticists are getting more and more concerned with respect to potential harmful effects of excessive Internet usage on our mental health (see for an overview an edited volume by Montag and Reuter, 2015a).

Although not an official diagnosis yet, the term *Internet addiction* has been coined 20 years ago by Young (1996, 1998a). Symptoms such as preoccupation with the Internet, withdrawal when not being online, development of tolerance, but also negative repercussions in one's own life due to excessive usage are emerging as important issues (e.g., Tao et al., 2010). Please note, that some researchers prefer the term problematic Internet use instead of Internet addiction, but the problem of terminological issues remains unresolved. In addition to these terms, others have put forward terms such as digital addiction (Thenu and Keerthi, 2013; Ali et al., 2015) or cyber addiction further complicating clear discussion of this issue (e.g., Billieux, 2012; Suissa, 2013).

Here we chose to use the term Internet addiction throughout the text, because it is most often used in the literature and seems the most straightforward. Indeed, mounting evidence from psychological and neuroscientific based studies is already providing support for some similiarities between substance-use disorders, such as alcoholism, and excessive Internet use fostering the idea that excessive Internet usage is indeed usefully characterized as a behavioral addiction. For instance, specific personality traits have been determined as vulnerabilitiy factors for both, Internet addiction as well as substance-use disorders, particulary low self-directedness and high neuroticism (e.g., Basiaux et al., 2001; Montag et al., 2010, 2011a; Sariyska et al., 2014). Moreover, neuroimaging research has determined common neurobiological alterations, including decreased gray matter volume/density in the anterior cingulate cortex (ACC) or exaggerated reactivity of the striatum to drug-related cues (Goldstein et al., 2009; Zhou et al., 2011; Montag et al., 2015a). In addition to associations between Internet addiction and problematic alcohol consumption (Ko et al., 2008; Yen et al., 2009), associations with other neuropathological disorders, particularly depression and attention deficit hyperactivity disorder (ADHD) have been reported (Young and Rogers, 1998; Ha et al., 2006; Yen et al., 2007; Sariyska et al., 2015). Thus, from various perspectives, overlaps between substance use disorders and Internet addiction have been observed. For instance, the mechanisms leading to the development and maintenance of Internet addiction share comparable aspects with other forms of addiction, sharing subcortical systems such as the dopamine mediated meso-limbic trajectory (Pierce and Kumaresan, 2006), well understood to mediate all drug addictions, as a shared substrate, but there are other perspectives. These will be further elaborated in the following theoretical framework.

Relying on findings such as those already noted, a growing number of theoretical frameworks have been proposed to understand Internet addiction. An important early framework stems from Davis (2001). At the heart of his framework is the classic idea of a stress-diathesis model, suggesting that a history of psychopathology combined with access to the Internet and positive reinforcement through the Internet could result in maladaptive cognitions such as “In the online-world I am a successful person, but in the offline-world I am a nobody”. Such potentially delusional thoughts are often reinforced by abundant online interactions (e.g., having success in online computer games or getting instant reward by funny or kind messages via online social communication channels such as Facebook or WhatsApp). This iterating mechanism can result either in a generalized form of Internet addiction or in distinct forms of excessive Internet usage in areas such as online social networks, Internet gaming, online shopping, online gambling or online pornography. The importance to distinguish among these different forms has already been supported by empirical evidence in a cross-cultural study (Montag et al., 2015b). Of note, recent developments in DSM-5 led to the inclusion of the term Internet Gaming Disorder in section III as an emerging disorder (Petry and O'Brien, 2013). Given the increasing evidence for diverse addictive behavior under the broad umbrella of “Internet addiction” perhaps a single category is a rather too narrow perspective.

Beside the described psychological theoretical framework of Davis (2001), other more neuroscientifically-based models have been put forward. A recent model from Brand et al. (2014) highlights dysfunctions in the fronto-striatal-limbic circuitry in Internet addiction, which might be key to understand the neurobiological basis of excessive Internet usage at a systems neuroscience level. When Internet addicts are confronted with Internet related cues, strong dopaminergic bursts originating from striatal regions along with impaired prefrontal top down regulation (impaired executive functions in the dorso-lateral prefrontal cortex and monitoring processes in the ACC) may gradually lead to a loss of control over Internet use. A new psychobiological model called I-PACE (Interaction of Person-Affect-Cognition-Execution) has been also put forward by Brand et al. (2016b), which will be focused upon in the context of our findings in the discussion. Dong and Potenza (2014) put forward an alternative model, but focused fairly narrowly on Internet Gaming Disorder, and will not be addressed in any detail in this paper; we refer the reader to the original manuscript by Dong and Potenza.

Although much is already known with respect to brain structures involved in Internet addiction, less is known about the molecular basis of the underlying brain (dys)functions. Some studies have already demonstrated some associations with molecular genetic markers (for an overview see Montag and Reuter, 2015a,b) and also psychopharmacological approaches have been put forward (see overviews Camardese et al., 2012, 2015). Among others, these studies yielded evidence for a role of dopaminergic and serotonergic systems in Internet addiction, and of course dopamine has been implicated in all addiction. For instance, psychopharmacological studies have found that administration of selective serotonin reuptake inhibitors (SSRIs) may help in the treatment of Internet addicted patients (Atmaca, 2007). In particular, a dopaminergic linkage with Internet addiction is getting most attention, because dopaminergic bursts in striatal regions have been found to accompany craving processes (and approach motivation toward all rewards, including drugs). This may lead to dopamine receptor downregulation, just as with alcohol addiction (Volkow et al., 2002), where lower dopamine_2_ receptor density has been observed in Internet addicts from positron emission tomography (PET) studies (Kim et al., 2011; Hou et al., 2012) as well as also from studies investigating the genetic make up of Internet addicts [Han et al., 2007; see also twin studies by Hahn et al. (2017) and Vink et al. (2015)]. In addition, another study revealed that a genetic variation on the CHRNA4 gene, which has been associated with trait anxiety and smoking, is also of relevance for Internet addiction (Montag et al., 2012a). This gene is a constituent of the cholinergic pathways of the brain.

Despite these initial findings, the molecular underpinnings of Internet addiction remain understudied and hence poorly understood. Thus neither a solid framework nor a clear roadmap for future studies is currently available. To this end, the present overview aims to provide such a framework, especially focussing on potential molecular mechanisms underlying the development and maintenance of Internet addiction. To promote such a framework, we will focus on two potentially most useful trajectories in the present overview.

First, we present data on how primary emotional systems can be linked to different facets of Internet addiction. Individual differences in primary emotional systems are assessed via a self-report questionnaire called *Affective Neuroscience Personaliy Scales* (ANPS) by Davis et al. (2003) in the present study. To our knowledge this questionnaire has yet to be utilized in investigation of Internet addiction. In contrast to classic questionnaires from personality psychology, which have been derived from a lexical approach (e.g., the prominent Five Factor Model of Personality/Big Five), the ANPS has been constructed on cross-species Affective Neuroscience (AN) studies of primary subcortical emotional systems (Panksepp, 1998b), which appear to be highly conserved across mammalian brains (Davis and Panksepp, 2011).

In brief, by means of deep electrical stimulation of the mammalian brain and by neurochemically specific pharmacological challenges, the AN approach has identified at least seven primary emotional systems, that have been labeled SEEKING, CARE, LUST, and PLAY (mediating positive emotions) and FEAR, SADNESS (aka PANIC), and ANGER (aka RAGE) (as the major negative emotions) which are driving mammalian unconditioned behaviors and associated affects and learning in a bottom up fashion. These ancient emotional circuitries represent tools for survival and have been extensively mapped with respect to their underlying brain systems (Panksepp, 1998b, 2005; Panksepp and Biven, 2012). Especially important for the present research endeavor, much is also known about their underlying neurotransmitter, especially specific neuropeptide activities.

Since the ANPS has not been investigated in the context of Internet addiction before, based on the current state of research, it is difficult to put forward specific hypotheses, in particular with respect to potential associations on symptom level such as mood regulation of the Generalized Problematic Internet Use Scale-2 (GPIUS-2). But given abundant research linking individual differences in positive/negative emotionality in terms of personality (e.g., extraversion or neuroticism) to Internet addiction (see for an overview the review by Montag and Reuter, 2015b), it can be expected that the positive emotions are inversely related with GPIUS-2 scores, whereas higher scores on the negative primary emotions should be associated with higher scores of the GPIUS-2.

Therefore, and secondly, the present study sought to apply the Affective Neuroscientific (AN) approach to understanding human emotions (Panksepp, 1998b) to the study of Internet addiction. This was done as follows: As described above, individual differences in primary emotional systems were assessed with the ANPS, while individual differences in Internet addiction were assessed with the Generalized Problematic Internet Use Scale-2 (GPIUS-2) developed by Caplan (2010). We decided to deploy the GPIUS-2 questionnaire to assess Internet addiction (instead of classic and important inventories such as Young's Internet addiction test, Young, 1998b), because the GPIUS-2 offers unique insights to distinct facets of problematic online usage behaviors such as (i) preference for online social interaction vs. real social interaction, (ii) cognitive preoccupation with the Internet, (iii) compulsive Internet use, and (iv) mood regulation by Internet usage or (v) negative outcomes due to overusage. The associations between primary emotional systems and different facets of Internet addiction were then used to sort primary mammalian brain emotional systems, as illuminated by direct studies of mammalian brains (Panksepp, 1998b) with various facets of Internet addiction.

## Materials and methods

### Participants

*N* = 680 participants (212 males, 468 females; age: *M* = 23.64, *SD* = 6.02) from the Ulm Gene Brain Behavior Project filled in the questionnaires ANPS and GPIUS-2. Most of the participants were students. All participants provided informed consent. The study was approved by the Ethics Committee of Ulm University, Ulm, Germany (information about the Ethics Committee is here: https://www.uni-ulm.de/einrichtungen/ethikkommission-der-universitaet-ulm.html).

### Questionnaires

The ANPS as published by Davis et al. (2003, also see Davis and Panksepp, 2011) consists of 110 items assessing six out of seven primary emotions. The positive emotions are SEEKING, CARE, PLAY, and the negative emotions are FEAR, SADNESS, and ANGER. LUST is not assessed, because here tendencies to answer in socially desirable ways might result in biased answers with potential carry-over effects to responses on the other scales. Each primary emotion was assessed with 14 items using a four point Likert scale ranging from totally disagree (1) to totally agree (4). An additional dimension is called Spirituality, which has been included due to its potential importance in the treatment of addiction. We do not focus on this scale, but report findings in the result section. The German version of the questionnaire has been used earlier (e.g., by Sindermann et al., 2016; in this study the ANPS has been investigated in the context of the 2D:4D marker as an indicator of prenatal testosterone and participants overlap to a great extent). Internal consistencies in the present sample were as follows: SEEKING α = 0.714, CARE α = 0.811, PLAY α = 0.803, FEAR α = 0.877, ANGER α = 0.816, SADNESS α = 0.737, Spirituality α = 0.846. SEEKING describes people who are interested in problem-solving, are open toward new experiences, like to explore new things and who are generally curious/inquisitive. CARE describes people who enjoy being with children and young pets, feel softhearted and like to care for others, especially sick ones. Also people high in CARE generally like the feeling of being needed by others. The PLAY scale is about having fun in comparison to being more serious-minded. It also captures if people like playing games with physical contact and enjoy humor and laughter. People scoring high on this scale are typically more playful, happy, and joyful. FEAR was defined as feeling anxious and tense, worrying a lot and ruminating on potentially harmful life problems, including tendencies to lose sleep because of worries and typically not being courageous. If a person scores high in SADNESS, the person is described as feeling lonely, thinking about loved ones/past relationships often as well as feeling distressed when alone. Typically these people tend to cry frequently. People scoring high in ANGER are typically hotheaded, easily irritated and frustrated (which often leads to feelings of anger, which may persist and be expressed verbally or physically). The Spirituality scale is about feeling connected to humanity and the creation as well as striving for inner peace and harmony (Davis et al., 2003).

The ANPS has been linked successfully to several biological variables including amygdala volumes (Reuter et al., 2009), molecular genetics (Felten et al., 2011; Montag et al., 2011b; Plieger et al., 2014), the 2D:4D marker as an indirect measure of prenatal testosterone (Sindermann et al., 2016) and also heritability estimates are readily available due to a recent twin study (Montag et al., 2016). Moreover, several recent studies also revealed the good psychometric properties (and stability) of the ANPS measure (Pingault et al., 2012; Geir et al., 2014; Orri et al., 2016). Several new studies used the ANPS also in clinical contexts (Farinelli et al., 2013; Karterud et al., 2016).

The GPIUS-2 by Caplan (2010) consists of 15 items assessing individual differences in Internet addiction. The reliability for the complete score consisting of all 15 items was α = 0.898 in the present study. Furthermore, three items always form one subscale with the following descriptors and with internal consistences reported in the brackets: preference for online social interaction (α = 0.830), mood regulation (α = 0.854), cognitive preoccupation (α = 0.726), compulsive Internet use (α = 0.877), negative outcomes (α = 0.872) (Caplan, 2010; p. 1093). We note that it is also possible to combine the scales compulsive Internet use and cognitive preoccupation to a factor called deficient self-regulation. For deeper insights into associations with the ANPS we present the more fine-granular data level. The German version has been used before by Montag et al. (2015b).

### Statistical analyses

Given the large sample size of the present sample, all statistical analyses were implemented using parametric tests (Bortz, 2005). First we report the influence of gender on the GPIUS-2 and the ANPS by using *T*-Tests. Furthermore, age was correlated with all variables using Pearson's correlations. In a subsequent step the GPIUS-2 and the ANPS were correlated. These correlations are also presented for males and females seperately. If age was associated with any of the variables, partial correlations were reported considering age as a control variable. Finally, hierarchical regression models were carried out to predict the overall GPIUS-2 scores and its subscales. In the course of these analyses we investigated the influence of age, gender (dummy coded: males “0,” females “1”) in a first block, which was followed by a second block where the relevant positive primary emotions were included. A third block followed with the relevant negative primary emotions. Relevant ANPS scales were all ANPS scales, which were significantly correlated with the respective GPIUS-2 scale in the whole sample. The rationale to insert the negative emotions in a third block was derived from the fact, that negative emotions play an important role in drug addictions (in particular late stages) and we anticipated that even after having considered socio-demography and positive primary emotions, negative affect should be able to explain an increment of the variance in the GPIUS-2 variables.

## Results

### Effects of gender and age on the GPIUS-2 and the ANPS scales

For the ANPS significant effects of gender were found on the scales CARE [*t*_(678)_ = −13.44, *p* < 0.001], FEAR [*t*_(678)_ = −7.41, *p* < 0.001], ANGER [*t*_(678)_ = −3.15, *p* = 0.002], SADNESS [*t*_(678)_ = −8.60, *p* < 0.001], and Spirituality [*t*_(678)_ = −2.63, *p* = 0.009]. Females scored higher on all of these ANPS scales. The scores of the overall GPIUS-2 scale [*t*_(678)_ = 3.63, *p* < 0.001] as well as the scales preference for online social interaction [*t*_(678)_ = 4.66, *p* < 0.001], compulsive Internet use [*t*_(678)_ = 2.98, *p* = 0.003], and negative outcomes [t_(678)_ = 5.10, *p* < 0.001] differed significantly between genders. In all of these scales males scored higher than females. The mean values and standard deviations of all scales for the whole sample as well as separately for males and females are presented in Tables 1, 2.

**Table 1:** **Means and standard deviations of the GPIUS-2 scales in the entire sample as well as split by gender**.

	**Entire sample**		**Males**		**Females**	
	***M***	***SD***	***M***	***SD***	***M***	***SD***
overall GPIUS-2 score	28.52	14.28	31.45	16.50	27.20	12.96
preference for online social interaction	5.06	3.40	5.95	4.18	4.66	2.90
mood regulation	8.40	5.39	8.67	5.47	8.28	5.35
cognitive preoccupation	4.94	2.99	5.19	3.41	4.83	2.78
compulsive Internet use	5.89	4.18	6.59	4.85	5.57	3.81
negative outcomes	4.23	2.87	5.05	3.92	3.86	2.13

**Table 2:** **Means and standard deviations of the ANPS in the entire sample as well as split by gender**.

	**Entire sample**		**Males**		**Females**	
	***M***	***SD***	***M***	***SD***	***M***	***SD***
SEEKING	39.84	4.42	39.60	4.58	39.95	4.35
CARE	41.19	5.97	37.13	5.93	43.04	5.01
PLAY	42.19	5.60	42.44	5.94	42.08	5.44
FEAR	36.58	6.67	33.87	6.30	37.81	6.47
ANGER	35.69	5.83	34.65	5.64	36.16	5.86
SADNESS	34.84	5.16	32.43	5.08	35.93	4.82
Spirituality	24.89	6.36	23.94	6.77	25.31	6.12

Age was significantly related to the ANPS scales CARE (*r* = −0.12, *p* = 0.001), PLAY (*r* = −0.19, *p* < 0.001), FEAR (*r* = −0.11, *p* = 0.006), SADNESS (*r* = −0.11, *p* = 0.005), and Spirituality (*r* = 0.11, *p* = 0.004) and the GPIUS-2 subscale mood regulation (*r* = −0.10, *p* = 0.011).

### Partial correlations between the GPIUS-2 and the ANPS

As correlations between age and several ANPS scales as well as one GPIUS-2 scale were found, all further correlational analyses were implemented using partial correlations. Age was implemented as a control variable.

Table 3 shows the partial correlations between the ANPS scales and the GPIUS-2 in the entire sample. The scale SEEKING of the ANPS was significantly negatively correlated to nearly all scales of the GPIUS-2, except mood regulation. The CARE scale was significantly negatively correlated with the overall GPIUS-2 score and the subscales preference for online social interaction, compulsive Internet use and negative outcomes. The PLAY scale of the ANPS was significantly negatively related to all scales of the GPIUS-2 except compulsive Internet use. FEAR was significantly positively associated with all GPIUS-2 scales. ANGER was significantly positively correlated with the overall GPIUS-2 score, preference for online social interaction, mood regulation and cognitive preoccupation. SADNESS was significantly positively related to all GPIUS-2 scales except the subscale negative outcomes (only trend significance was observed here, *r* = 0.08). Spirituality was not correlated to any of the GPIUS-2 scales. Most of the correlation (especially regarding the scales SEEKING and FEAR) mentioned as significant remain significant even after correcting for multiple testing (0.05/42 = 0.00119).

**Table 3:** **Partial correlations between the ANPS and the GPIUS-2 scales corrected for age in the entire sample**.

	**Overall GPIUS-2 score**	**Preference for online social interaction**	**Mood regulation**	**Cognitive preoccupation**	**Compulsive Internet use**	**Negative outcomes**
SEEKING	*r* = −0.16^***^	*r* = −0.16^***^	*r* = −0.05	*r* = −0.13^***^	*r* = −0.13^***^	*r* = −0.20^***^
CARE	*r* = −0.14^***^	*r* = −0.22^***^	*r* = −0.03	*r* = −0.07	*r* = −0.08^*^	*r* = −0.18^***^
PLAY	*r* = −0.17^***^	*r* = −0.27^***^	*r* = −0.09^*^	*r* = −0.11^**^	*r* = −0.05	*r* = −0.16^***^
FEAR	*r* = 0.24^***^	*r* = 0.16^***^	*r* = 0.24^***^	*r* = 0.22^***^	*r* = 0.17^***^	*r* = 0.11^**^
ANGER	*r* = 0.13^***^	*r* = 0.11^**^	*r* = 0.12^**^	*r* = 0.17^***^	*r* = 0.05	*r* = 0.05
SADNESS	*r* = 0.23^***^	*r* = 0.10^*^	*r* = 0.28^***^	*r* = 0.24^***^	*r* = 0.13^***^	*r* = 0.08
Spirituality	*r* = 0.02	*r* = −0.01	*r* = −0.03	*r* = 0.07	*r* = 0.03	*r* = 0.04

*All reported p-values are two-tailed*.

*** *p <= 0.001*,

** *p <= 0.01*,

* *p <= 0.05*.

As seen in Table 4, in the male sample SEEKING and the GPIUS-2 scales were robustly negatively linked. Only the correlation between SEEKING and mood regulation did not reach significance. The only significant correlation between the CARE scale and the GPIUS-2 was a negative correlation with the subscale preference for online social interaction. The PLAY scale was significantly negatively related to all GPIUS-2 scales. Regarding the associations between FEAR and all GPIUS-2 scales, all correlations were significant and positive. Of note, all these correlations remain significant even after correction for multiple testing (0.05/42 = 0.00119) except the correlation with the scale negative outcomes. The ANGER scale is positively related to the overall GPIUS-2 score and the GPIUS-2 subscales preference for online social interaction, cognitive preoccupation and compulsive Internet use. The correlation between ANGER and the subscale negative outcomes failed to be significant (*p* = 0.13). The same was true for the association with mood regulation (*p* = 0.11). The SADNESS scale is significantly positively related to all GPIUS-2 scales. Again the Spirituality scale is not significantly correlated to any of the GPIUS-2 scales.

**Table 4:** **Partial correlations between the ANPS and the GPIUS-2 scales corrected for age in the male subsample**.

	**Overall GPIUS-2 score**	**Preference for online social interaction**	**Mood regulation**	**Cognitive preoccupation**	**Compulsive Internet use**	**Negative outcomes**
SEEKING	*r* = −0.27^***^	*r* = −0.22^***^	*r* = −0.12	*r* = −0.18^**^	*r* = −0.21^**^	*r* = −0.31^***^
CARE	*r* = −0.10	*r* = −0.17^*^	*r* = −0.03	*r* = −0.08	*r* = −0.02	*r* = −0.11
PLAY	*r* = −0.26^***^	*r* = −0.32^***^	*r* = −0.15^*^	*r* = −0.15^*^	*r* = −0.16^*^	*r* = −0.23^***^
FEAR	*r* = 0.37^***^	*r* = 0.22^***^	*r* = 0.36^***^	*r* = 0.29^***^	*r* = 0.29^***^	*r* = 0.21^**^
ANGER	*r* = 0.20^**^	*r* = 0.21^**^	*r* = 0.11	*r* = 0.18^**^	*r* = 0.15^*^	*r* = 0.13
SADNESS	*r* = 0.32^***^	*r* = 0.18^**^	*r* = 0.32^***^	*r* = 0.27^***^	*r* = 0.24^***^	*r* = 0.16^*^
Spirituality	*r* = 0.04	*r* = 0.07	*r* = −0.04	*r* = 0.08	*r* = 0.04	*r* = 0.04

*All reported p-values are two-tailed*.

*** *p <= 0.001*,

** *p <= 0.01*,

* *p <= 0.05*.

As seen in Table 5, the SEEKING scale is significantly negatively related to all GPIUS-2 scales except mood regulation and compulsive Internet use in the female sample. The CARE scale is only significantly negatively related to preference for online social interaction and negative outcomes. The PLAY scale of the ANPS is significantly negatively related to the overall GPIUS-2 score, preference for online social interaction, cognitive preoccupation and negative outcomes. The FEAR and SADNESS scales of the ANPS are significantly positively related to all GPIUS-2 scales. All of the correlations between the FEAR scale and the GPIUS-2 scales and most of the correlations between SADNESS and the GPIUS-2 scales would remain significant even after correction for multiple testing (0.05/42 = 0.00119). ANGER is significantly positively related to the overall GPIUS-2 score, preference for online social interaction, mood regulation and cognitive preoccupation. At last the Spirituality scale is only significantly positively related to the subscale negative outcomes of the GPIUS-2.

**Table 5:** **Partial correlations between the ANPS and the GPIUS-2 scales corrected for age in the female subsample**.

	**Overall GPIUS-2 score**	**Preference for online social interaction**	**Mood regulation**	**Cognitive preoccupation**	**Compulsive Internet use**	**Negative outcomes**
SEEKING	*r* = −0.09^*^	*r* = −0.12^*^	*r* = −0.01	*r* = −0.09^*^	*r* = −0.08	*r* = −0.12^*^
CARE	*r* = −0.07	*r* = −0.16^***^	*r* = −0.01	*r* = −0.02	*r* = −0.04	*r* = −0.10^*^
PLAY	*r* = −0.13^**^	*r* = −0.27^***^	*r* = −0.06	*r* = −0.10^*^	*r* = 0.00	*r* = −0.13^**^
FEAR	*r* = 0.26^***^	*r* = 0.21^***^	*r* = 0.21^***^	*r* = 0.22^***^	*r* = 0.18^***^	*r* = 0.15^***^
ANGER	*r* = 0.13^**^	*r* = 0.10^*^	*r* = 0.14^**^	*r* = 0.17^***^	*r* = 0.02	*r* = 0.04
SADNESS	*r* = 0.28^***^	*r* = 0.14^**^	*r* = 0.30^***^	*r* = 0.27^***^	*r* = 0.14^**^	*r* = 0.15^***^
Spirituality	*r* = 0.03	*r* = −0.03	*r* = −0.02	*r* = 0.07	*r* = 0.05	*r* = 0.10^*^

*All reported p-values are two-tailed*.

*** *p <= 0.001*,

** *p <= 0.01*,

* *p <= 0.05*.

In conclusion, over all samples, as well as the male and female only sample, the ANPS scales of positive affect (SEEKING, CARE, PLAY) are negatively linked to most of the GPIUS-2 scales. In contrast, the ANPS scales of negative affect (FEAR, ANGER, SADNESS) are positively linked to most the GPIUS-2 scales across genders.

### Stepwise regressions

In a next step, stepwise regression analyses were implemented. Thereby the amount of explained variance in the GPIUS-2 scales through age, gender, and the ANPS scales, were examined. Results for the overall GPIUS-2 score as well as the subscales are presented in Tables 6–11.

**Table 6:** **Hierarchical regression model for the overall GPIUS-2 score**.

	**β**	***T***	***p***	**Δ*R*^2^**	***R*^2^**
Age	−0.070	−1.89	0.060		
Gender	−0.201	−4.80	<0.001	0.025	0.025
SEEKING	−0.072	−1.86	0.064		
CARE	−0.120	−2.64	0.009		
PLAY	−0.014	−0.32	0.746	0.042	0.067
FEAR	0.145	2.80	0.005		
ANGER	0.036	0.93	0.351		
SADNESS	0.208	4.03	<0.001	0.084	0.150

*Variables included in the regression analysis were age, gender and all scales of the ANPS except the scale Spirituality*.

**Table 7:** **Hierarchical regression model for the GPIUS-2 subscale preference for online social interaction**.

	**β**	***T***	***p***	**Δ*R*^2^**	***R*^2^**
Age	−0.014	−0.39	0.699		
Gender	−0.181	−4.30	<0.001	0.031	0.031
SEEKING	−0.038	−0.98	0.326		
CARE	−0.110	−2.39	0.017		
PLAY	−0.188	−4.27	<0.001	0.087	0.118
FEAR	0.097	1.86	0.063		
ANGER	0.064	1.67	0.095		
SADNESS	0.047	0.90	0.367	0.021	0.140

*Variables included in the regression analysis were age, gender and all scales of the ANPS except the scale Spirituality*.

**Table 8:** **Hierarchical regression model for the GPIUS-2 subscale mood regulation**.

	**β**	***T***	***p***	**Δ*R*^2^**	***R*^2^**
Age	−0.083	−2.19	0.029		
Gender	−0.156	−4.04	<0.001	0.012	0.012
PLAY	0.003	0.09	0.932	0.008	0.020
FEAR	0.095	1.83	0.067		
ANGER	0.025	0.66	0.511		
SADNESS	0.257	5.03	<0.001	0.093	0.114

*Variables included in the regression analysis were age, gender and the ANPS scales PLAY, FEAR, ANGER and SADNESS*.

**Table 9:** **Hierarchical regression model for the GPIUS-2 subscale cognitive preoccupation**.

	**β**	***T***	***p***	**Δ*R*^2^**	***R*^2^**
Age	−0.026	−0.68	0.499		
Gender	−0.150	−3.82	<0.001	0.004	0.004
SEEKING	−0.076	−1.92	0.055		
PLAY	−0.010	−0.25	0.805	0.022	0.026
FEAR	0.080	1.51	0.130		
ANGER	0.093	2.39	0.017		
SADNESS	0.191	3.69	<0.001	0.072	0.098

*Variables included in the regression analysis were age, gender and the ANPS scales SEEKING, PLAY, FEAR, ANGER and SADNESS*.

**Table 10:** **Hierarchical regression model for the GPIUS-2 subscale compulsive Internet use**.

	**β**	***T***	***p***	**Δ*R*^2^**	***R*^2^**
Age	−0.063	−1.67	0.096		
Gender	−0.168	−3.89	<0.001	0.018	0.018
SEEKING	−0.077	−1.95	0.052		
CARE	−0.045	−1.02	0.306	0.017	0.034
FEAR	0.156	2.99	0.003		
SADNESS	0.078	1.48	0.139	0.039	0.074

*Variables included in the regression analysis were age, gender and the ANPS scales SEEKING, CARE, FEAR and SADNESS*.

**Table 11:** **Hierarchical regression model for the GPIUS-2 scale negative outcomes**.

	**β**	***T***	***p***	**Δ*R*^2^**	***R*^2^**
Age	−0.096	−2.51	0.012		
Gender	−0.204	−4.77	<0.001	0.043	0.043
SEEKING	−0.133	−3.35	0.001		
CARE	−0.073	−1.61	0.107		
PLAY	−0.059	−1.33	0.183	0.050	0.093
FEAR	0.120	2.84	0.005	0.011	0.104

*Variables included in the regression analysis were age, gender and the ANPS scales SEEKING, CARE, PLAY and FEAR*.

Gender had a significant effect on all GPIUS-2 scales with males showing higher scores compared to females. Moreover, even after the positive primary emotions were already included in the model, in a second step, the negative primary emotions still explained a significant part of variability in most of the GPIUS-2 scales when included in a third step. Only in the regression model for the GPIUS-2 scale preference for online social interaction, none of the negative primary emotions explained a significant part of the variance over age, gender, and the positive primary emotions. In summary, especially the scale FEAR and also SADNESS are the ANPS scales most strongly associated with nearly all (sub)scales of the GPIUS-2. Both are positively associated with the corresponding GPIUS-2 (sub)scales.

## Discussion

### General discussion

To our knowledge the present study investigates for the first time how individual differences in primary emotional systems as assessed by the ANPS relate to individual differences in tendencies toward Internet addiction. Considering associations between individual differences in the ANPS and the total score of the GPIUS-2, it becomes obvious that higher scores in all negative primary emotional systems (FEAR, SADNESS, ANGER) are robustly linked to higher tendencies toward problematic usage of the Internet, while reverse results are observed for all positive emotional systems. In addition, overall Internet addiction scores could be best predicted by higher scores of both the FEAR and SADNESS systems, or by lower scores in the CARE system. This underlines the already described associations between Internet addiction and depression (see Sariyska et al., 2015), but also links between Internet addiction and neuroticism (e.g., Hardie and Tee, 2007; Montag et al., 2010). It has been discussed (Davis and Panksepp, 2011; Montag, 2014) that individual differences in primary emotional systems could represent the evolutinary oldest parts of human personality and FEAR/SADNESS seem to be robustly associated with neuroticism (see Montag et al., 2013; Sindermann et al., 2016).

Determining distinct facets of excessive Internet use is of importance for both, neurobiological research as well as the clinical practice. Notably, in the present study distinct facets of Internet addiction are differently associated with the primary emotional systems as assessed by the ANPS. High preferences for online social interaction seem best predicted by low PLAY scores. Although the present study cannot provide insights into causal mechanisms such as that low PLAY scores are potentially a predisposition for or a consequence of Internet addiction, the findings are noteworthy for identifying possible relevant personality dispositions. In our view the negative link between PLAY and the preference for online social interaction is intriguing in the light of (i) the debate of possible associations between Internet addiction and ADHD (Yoo et al., 2004; Sariyska et al., 2015), and (ii), as well as the possibility of diminished early social play in children eventually diagnosed with ADHD (Panksepp, 1998a, 2008). Indeed, animal studies yielded preliminary evidence that a lack of rough and tumble play in young animals may lead to ADHD symptoms (Panksepp et al., 2003). A corrolary of this may be that excessive Internet use in children may lead to decreased real-world social play, which may in turn promote the development of ADHD symptoms. These interactions between excessive Internet use and the development of ADHD might be further explored in future studies (obviously causal-linkages cannot be identified from cross-sectional correlational studies such as the present one).

Considering the many facets of mood regulation and preoccupation with the Internet, it is noteworthy that SADNESS scores were one of the best predictors for Internet addiction as monitored with GPIUS-2. Therefore, individuals scoring high on the SADNESS personality dimension may use the Internet as a social-surrogate for mood upregulation, perhaps especially when feeling emotionally “down” or upset as indicated by the relevant items of the GPIUS-2 (Caplan, 2010; p. 1093). Accordingly, the present findings could be interpreted in two ways: (i) Individuals with high SADNESS may want to downregulate their negative emotionality by more persistent Internet use as compared to more modest Internet users; (ii) alternatively, it is possible that higher SADNESS among the primary emotional system may be a long-term consequence of Internet overusage. Since the ANPS measures long-term traits and not short-term states, and personality traits are deemed rather stable over extended time (Edmonds et al., 2008; Orri et al., 2016), we suggest the first explanation may be the more appropriate one. Of course, this would need to be evaluated with a longitudinal design.

Finally, let us consider the facets of compulsive Internet use and negative outcomes due to Internet overusage: Compulsive Internet use probably reflects loss of control with respect to one's own Internet overusage. Indeed, high Internet use scores are best predicted by high FEAR scores, which suggests that chronic high anxiety might be at the heart of compulsive usage. Further, negative outcomes can be best predicted by low SEEKING scores, suggesting the hypothesis that i) either low SEEKING scores are a negative affective outcome of Internet overuse or ii) that low SEEKING, as a primary (constitutional) emotional trait, which could be expected to diminish gregariousness, predisposes individuals to exhibit escalated interaction with inanimate objects (where they have full control) yielding Internet addiction (which can be described as a negative outcome, at least from external perspectives). A final note: The ANPS FEAR scale is mostly designed to assess mild anxiety and not intense fear. For additional discussions and measures for disentangling anxiety and fear, see (Markett et al., 2014; Reuter et al., 2015).

### Toward a molecular understanding of internet addiction

In the debate about Internet addiction and its inclusion in the upcoming ICD-11 much research has been conducted in classic psychology and the neurosciences providing support for the view that overusage of the Internet may indeed be well characterized as a behavioral addiction (see overviews Brand et al., 2014; Montag et al., 2015a). In the neurosciences the most prominent evidence for understanding Internet addiction has come from magnetic resonance imaging (MRI) and to a lesser extent electroencephalography (EEG) and positron emission tomography (PET) studies (e.g., Kim et al., 2011; see also overview by Montag et al., 2015a). To date, direct evidence for the molecular underpinnings of Internet addiction remains sparse (with the exception of the few above noted studies from molecular genetics and psychopharmacology). Aside from such suggestive work, a theoretical framework that can provide a coherent roadmap for the study of the molecular underpinnings of Internet addiction is not available yet. Therefore, we would like to use the results from the present study using self-report measures assessing individual differences in Internet addiction and primary emotional traits to help establish a preliminary model about which brain areas, and more importantly, *which underlying neurotransmitters* may currently help illuminate a scientific understanding of Internet addiction. The usefulness of such an approach has been recently deployed to demonstrate how emotional facial actions, illuminated by Paul Ekman's work, can be integrated with the Affective Neuroscience theoretical framework to study the brain molecular/neurotransmitter basis of human affective expressions (Montag and Panksepp, 2016). Such ideas have already been put forward as workable ideas in the area of personality psychology (Montag and Reuter, 2014).

We provide a detailed roadmap (i.e., working hypotheses) for such ideas in Table 12, where we note the current most likely strongest associations between the various distinct facets of Internet addiction (as assessed by the GPIUS-2) and the most likely, (i.e., currently most highly-relevant) primary emotional system(s). Namely, on the left side of Table 12 the subscales of GPIUS-2 mirroring (some, but not all) important symptoms of Internet addiction are presented together, with their closely linked primary emotional networks as derived from the present questionnaire study. On the right side the neuroanatomical structures including the relevant neurotransmitter/neuropeptide systems are summarized either activating or inhibiting each neuronal circuitry underlying the distinct primary emotional systems. Again, this is possible, as the primary emotional systems have been mapped in detail with respect to their neuroanatomy and underlying neurotransmitter/neuropeptides. The ANPS has been constructed on the background of these data (see for overviews see Panksepp, 1998b, 2011).

**Table 12:** **A synopsis of the cross-species primary emotional systems and their underlying neuroanatomical structures and neurotransmitter/neuropeptides (information taken from Panksepp, 1998b, 2011; Montag and Panksepp, 2016)**.

**Distinct facets of Internet addiction**	**Panksepp's primary emotional systems**	**Brain neuroanatomy related to these primary emotional systems**	**Neuropeptides/neurotransmitters related to these primary emotional systems**
Overall GPIUS-2 score	FEAR	Central and lateral amygdala to medial hypothalamus and dorsal periaqueductal gray (PAG)	Glutamate (+), CRF(+), CCK(+), alpha-MSH(+), oxytocin (—)
Preference for online social interaction			
Compulsive Internet use			
Negative outcomes			
Cognitive Preoccupation	RAGE	Medial amygdala to bed nucleus of stria terminalis (BNST). Medial and perifornical hypothalamus to PAG	Substance P (+), Ach (+), glutamate (+)
Overall GPIUS-2 score	PANIC/SADNESS	Anterior cingulate, BNST and preoptic area, dorsomedial thalamus, PAG	Opioids (−), oxytocin (−), prolactin (−), CRF (+), glutamate (+)
Mood regulation			
Cognitive Preoccupation			
Negative Outcomes	SEEKING	Nucleus accumbens—ventral tegmental area (VTA), mesolimbic and mesocortical outputs, lateral hypothalamus—PAG	Dopamine (+), glutamate (+), opioids (+), neurotensin (+), orexin (+)
Overall GPIUS-2 score	CARE	Anterior cingulate, BNST, preoptic area, VTA, PAG	Oxytocin (+), prolactin (+), dopamine (+), opioids (+/−)
	LUST	Cortico-medial amygdala, BNST, preoptic hypothalamus, ventromedial hypothalamus (VMH), PAG	Gonadal steroids (+), vasopressin (+ male), oxytocin (+ female), LH-RH (+)
Preference for online social interaction	PLAY	Dorso-medial diencephalon, parafascicular area, PAG	Opioids (+/−), glutamate (+), Ach (+), endocannabinoids (+)

*+ Excitatory effects/− inhibiting effects; CRF: corticotropin releasing factor/hormone, CCK: cholecystokinin, alpha-MSH: alpha melanocyte stimulating hormone, Ach: acetylcholine, LH-RH: luteinising hormone releasing hormone. The potential LUST association with the GPIUS-2 is presented below the CARE circuitry, because both LUST and CARE are deeply entwined. Of note, LUST has not been assessed with the ANPS. The present potential association is only presented as an idea for future research projects*.

By linking cross-species Affective Neuroscience approaches with the study of Internet addiction, a coherent framework emerges, which may eventually enable investigators to test several brain molecular candidates that may help us to better characterize and understand Internet addiction. Such integration may also facilitate development of treatments for the various facets of Internet addiction. Here, we would like to add an important issue. A recent new model called I-PACE (Interaction of Person-Affect-Cognition-Execution) was published to explain that the genesis of Internet addiction can be further clarified by the aforementioned interaction of variables (Brand et al., 2016b). Our framework can be integrated in this model, as the I-PACE includes the biopsychological consitution of a person representing a resilience or vulnerability factor for Internet addiction.

In detail, the I-PACE model of specific Internet-use disorders represents a process model, which integrates biological (e.g., genetics) as well as psychological characteristics (e.g., early childhood experiences) of a person as vulnerability factors. These predisposing factors are thought to interact with other moderating factors like for example coping styles or Internet related biases. According to the model, the co-occurrence and interactions of unfavorable factors lead to a situation in which—from the cognitive and emotional perspective of a person—using the Internet is favorable. If the use of the Internet is perceived as a gratification, the internal psychological patterns (e.g., biases) can lead to an understanding of how the compulsive use of the Internet is reinforced. This strengthens the use of the Internet in similar future situations resulting in maladaptive behavioral patterns.

With loss of control over the Internet use and emerging negative consequences for daily live, a specific Internet-use disorder develops. As Brand et al. (2016b) mentioned, by considering genetic underpinnings as predisposing factors for the emergence of specific Internet-use disorders, the results of the present study (with special regard to Table 12) can be used to build specific directed-hypotheses about which molecules or molecular genetic underpinnings contribute to the emergence of specific Internet-use disorders. For example, the neuronal circuitry for SADNESS is known to be downregulated by the neuropeptide oxytocin (Panksepp, 1998b). As SADNESS has been linked with the overall GPIUS-2 score and its subfacets such as mood regulation in the present study, (low) oxytocin levels could be a key predisposing factor for the emergence of Internet addiction on endocrinological, but also molecular genetic level. For instance, low empathy, with possible links to oxytocin, is associated with higher Internet addiction (Melchers et al., 2015). Thus, oxytocin is an interesting candidate to be tested in future work (also in the context of the I-PACE model). Moreover, we, as well as Brand et al. (2016b), have already noted that affective responses play an important role, when a person hooked to the Internet is confronted with an Internet related cue. Primary emotional systems may causally elicit relevant emotional reactions. Hence, from this perspective, the present study may help relate the I-PACE model with life circumstances. To foster such connections, we would like to give an example on how Table 12 may be used in general. We demonstrated above that low CARE, and high SADNESS / FEAR predict overall Internet addiction. Given that the neuropeptide oxytocin plays an important role in the neuronal circuitry underlying CARE (facilitation) and SADNESS (inhibition), but also the FEAR dimension (inhibition), the adminsitration of oxytocin might help to alter these circuitries to reduce SADNESS and FEAR, while strengthening CARE and exploration, creativity and extraverted openness to experience (De Dreu et al., 2015) with the consequence of engaging increasingly in interactions with persons in “real” life while simultaniously reducing online social interactions.

In this context it is noteworthy that oxytocin has been found to attenuate autistic symptoms (Hollander et al., 2007; Guastella et al., 2010) and facilitate emotion recognition (Domes et al., 2007). As Internet addiction also has been associated with low empathy (Melchers et al., 2015), oxytocin might improve social cognition in face-to-face interactions in preference to less personal online social discourse. Further, with the ANPS, one can also link various primary emotional system strengths and weaknesses to specific facets of Internet addiction (and not just overall GPIUS-2 scores). For example, as the SADNESS dimension is linked to the facet of mood regulation and cognitive preoccupation, the administration of oxytocin might in particular have positive therapeutic effect on these facets of Internet addiction. For some preliminary empirical evidence on a link between oxytocin and Internet addiction see the genetic association reported between variation of the OXTR gene and Internet addiction in the conference paper by Sariyska et al. (2016).

Some limitations need to be considered. First of all, the present theoretical framework has been derived from a study using questionnaires without the assessment of biological variables in the present participants. Moreover, self-report assessment of one's own primary emotional systems is an indirect approach to one's own emotional world—in a way it is a cognitive approach to our emotions. Davis and Panksepp (2011; p. 1952) state it as follows: “we interpret the ANPS scales as tertiary (thought-mediated) approximations of the influence of the various primary emotional systems in people's lives”. Development of more direct measures of emotional activity is clearly of high relevance. Another concern relates to the diverse neuroscientific frameworks highlighting the tremendous relevance of neocortical brain areas such as the dorso-lateral prefrontal cortex and medial prefrontal cortex—the “seats” of executive functioning and emotion regulation in the human brain (Levy and Goldman-Rakic, 2000). Clearly, our roadmap to the molecular study of Internet addiction is limited, as we here targeted only the emotional side of this disorder. Various cognitive-style facets will need to be integrated into future work. Another issue arises from evidence in Table 12: ANGER was not strongly linked in our data set to the assessed facets of Internet addiction, although some significant correlations appeared, which seemed more straightforwardly linked to overlaps with other primary emotional systems (of further note: LUST was not assessed since it is not included in the ANPS). Nevertheless, as noted earlier, it may be of interest/importance to assess addictive tendencies of patients in distinct areas of Internet usage such as online pornography addiction, which may relate intimately to LUST circuitry (e.g., Brand et al., 2016a). In addition, Internet Gaming Disorder may have linkages to online first-person-shooter-video games (Montag et al., 2011a), which may be linked to excessive arousal of the RAGE/irritability personality dimension (Montag et al., 2012b).

Finally, we would reflect briefly on some new emerging disorder of the modern electronic communication age—the smartphone addiction (for additional information on smartphone usage and personality, see Montag et al., 2015c). As also outlined by Kwon et al. (2013a,b) the overlap between Internet and smartphone addiction hovers around correlations of 0.50 (hence 25% of shared variance), suggesting that the conceptual roadmap summarized here may be transferable, to some extent, to the overusage of other interactive electric media, especially smartphones. For further discussions of how an ANPS approch to personality assessment may represent an interesting framework for the neuroscientific study of Internet/smartphone addiction see Montag and Walla (2016). Indeed, we have already collected some data on smartphone addiction from all participants; accordingly, we share correlation patterns between smartphone addiction and the ANPS in Table 13 of this paper. This allows readers to apply the same strategy as described in Table 12 to establish hypothesis on the molecular basis of smartphone addiction. As with Internet addiction, both FEAR and SADNESS show the highest correlations with smartphone addiction scores. As Internet addiction and smartphone addiction share 24% of the variance in our data set (correlation of *r* = 0.49 between the overall GPIUS-2 and the Smartphone Addiction Scale (SAS) score), the SADNESS and FEAR associations seem to fall in the shared variance of smartphone / Internet addiction correlations. The SAS has been originally published by Kwon et al. (2013b). The internal consistencies for the present questionnaire data are as follows: total SAS score α = 0.995, daily-life disturbance α = 0.841, positive anticipation α = 0.874, withdrawal = 0.829, cyberspace-oriented relationship α = 0.826, overuse α = 0.754, tolerance α = 0.823.

**Table 13:** **Partial correlations between the ANPS and the Smartphone Addiction Scale corrected for age in the entire sample**.

	**Total SAS score**	**Daily-life disturbance**	**Positive anticipation**	**Withdrawal**	**Cyberspace-oriented relationship**	**Overuse**	**Tolerance**
SEEKING	*r* = −0.13^***^	*r* = −0.12^***^	*r* = −0.08^*^	*r* = −0.14^***^	*r* = −0.14^***^	*r* = −0.09^*^	*r* = −0.10*^*^
CARE	*r* = −0.07	*r* = −0.02	*r* = −0.05	*r* = −0.09^*^	*r* = −0.08^*^	*r* = −0.05	*r* = −0.03
PLAY	*r* = −0.07	*r* = −0.04	*r* = −0.03	*r* = −0.09^*^	*r* = −0.12^**^	*r* = −0.03	*r* = −0.04
FEAR	*r* = 0.17^***^	*r* = 0.18^***^	*r* = 0.11^**^	*r* = 0.16^***^	*r* = 0.15^***^	*r* = 0.15^***^	*r* = 0.12^**^
ANGER	*r* = 0.12^**^	*r* = 0.10^**^	*r* = 0.06	*r* = 0.15^***^	*r* = 0.07	*r* = 0.14^***^	*r* = 0.13^***^
SADNESS	*r* = 0.13^***^	*r* = 0.15^***^	*r* = 0.08^*^	*r* = 0.12^**^	*r* = 0.15^***^	*r* = 0.11^**^	*r* = 0.10^*^
Spirituality	*r* = 0.08^*^	*r* = 0.10^**^	*r* = 0.07	*r* = 0.04	*r* = 0.06	*r* = 0.06	*r* = 0.10^**^

*All reported p-values are reported two-tailed*.

*** *p <= 0.001*,

** *p <= 0.01*,

* *p <= 0.05*.

## Conclusion

The present study demonstrated the usefulness of the ANPS to understand individual differences in Internet addiction. Considering correlations between the ANPS and the GPIUS-2, this work provides the first roadmap for the molecular study of Internet addiction. We believe that the present work, although offering a new personality and theoretical framework, is further enriched, by being connected with already existing models such as the I-PACE.

## Author contributions

CM and JP designed the study and wrote the protocol. Author CM did the literature research, Author CS carried out the statistical analyses, and the formatting of the manuscript. Authors CM and CS wrote the manuscript. Author BB gave further insights and checked the whole manuscript. Also author JP worked over the first and revised draft of the manuscript, provided additional valuable insights and checked the manuscript.

## Funding

The position of CM is funded by a Heisenberg grant awarded to him by the German Research Foundation (DFG, MO 2363/3-1). Moreover, the study was funded by a grant of the German Research Foundation (DFG MO2363/2-1) awarded to CM to study the biological basis of Internet addiction.

### Conflict of interest statement

The authors declare that the research was conducted in the absence of any commercial or financial relationships that could be construed as a potential conflict of interest.

## Footnotes

^1^www.internetworldstats.com/stats.htm
